# Intelligent Sports Weights

**DOI:** 10.3390/s25123808

**Published:** 2025-06-18

**Authors:** Olga dos Santos Duarte, Gustavo Jacinto, Mário Véstias, Rui Policarpo Duarte

**Affiliations:** 1 Instituto Superior de Engenharia de Lisboa, Instituto Politécnico de Lisboa, Rua Conselheiro Emidio Navarro, 1, 1959-007 Lisboa, Portugal; a27675@alunos.isel.pt (O.d.S.D.); a46006@alunos.isel.pt (G.J.); mario.vestias@isel.pt (M.V.); 2 INESC INOV, 1000-029 Lisboa, Portugal; 3 Instituto Superior Técnico, University of Lisbon, Av. Rovisco Pais, 1, 1000-0001 Lisboa, Portugal

**Keywords:** intelligentfitness, human activity recognition, low-power, embedded IoT

## Abstract

Weightlifting is a common fitness activity and can be practiced individually without supervision. However, performing regular weightlifting exercises without any form of feedback can lead to serious injuries. To counter this, this work proposes a different approach to automatic weightlifting supervision off-the-person. The proposed embedded system is coupled to the weights and evaluates if they follow the correct trajectory in real time. The system is based on a low-power embedded System-on-a-Chip to perform the classification of the correctness of physical exercises using a Convolutional Neural Network with data from the embedded IMU. It is a low-cost solution and can be adapted to the characteristics of specific exercises to fine-tune the performance of the athlete. Experimental results show real-time monitoring capability with an average accuracy close to 95%. To favor its use, the prototypes have been enclosed on a custom 3D case and validated in an operational environment. All research outputs, developments, and engineering models are publicly available.

## 1. Introduction

The ability to survey the quality of physical exercise execution is crucial to achieve faster and consistent results from training. Exercising without expert guidance can result in incorrect exercise performance, thus increasing the risk of injuries. Additionally, elite sports can benefit from a real-time classification system to condition the execution of exercises to follow pre-trained custom exercises. Since most athletes already have accessories on them and do not welcome having to add new ones, it is of great importance to have a small and low-cost system capable of classifying the quality of the exercises off-the-person in any environment.

Most of the existing movement classification systems rely on image acquisition, which is very sensitive to changes in scenery and lighting and difficult to set up. The availability of compact autonomous low-power embedded systems with built-in sensors has enabled the creation of very compact smart sensors integrated into gym equipment, providing real-time feedback on exercise movements. However, there are systems around a similar architecture, but they are used only to classify which is the most similar exercise performed, not the correctness. In any system, the correctness of the exercise is supervised by a professional trainer.

This paper presents a novel system for automatically recognizing the correctness of movements in physical exercise using Machine Learning algorithms on low-cost and low-power Microcontroller Units (MCUs) with integrated movement sensors. The system is demonstrated while being coupled to a weight via magnetic attraction. Moreover, by having the system on the weight (dumbbell, barbell, or kettlebell) and not on the wrist of the person, it is able to capture the contributions of the wrist movement, which systems on the person cannot perform.

The proposed system is capable of real-time sensor data acquisition, processing the data using trained neural networks to classify the exercise execution quality, and wirelessly communicating this information with a host computer or mobile phone. It has been validated on the operational environment, which is equivalent to a Technology Readyness Level (TRL) of 6.

To facilitate replication of the results and innovation upon this work, all sources, including the 3D case, are made publicly available under an open-source MIT License.

## 2. Background and Related Work

Human Activity Recognition (HAR) research is focused on developing systems to automatically recognize and categorize human actions based on sensor data, interpreting body movements to determine activities [1]. It is used to analyze sports’ performance by tracking the athletes’ movements to anticipate any injury risks and assessing training programs [2].

Machine Learning (ML) has been used in the creation of models from data for classification in low-power devices [3,4], while Tiny Machine Learning (TinyML) refers to deploying ML models on resource-constrained devices, and TensorFlow Lite (TFLite) is optimized for running ML models in limited-resource devices [5].

Convolutional Neural Networks (CNNs) are commonly used for HAR systems, combining feature extraction and classification from raw time-series data. The architecture discussed in [6] features parallel branches for temporal convolutions and max-pooling operations to create intermediate representations for classification. The results highlight the benefits of using max-pooling and varying learning rates during training.

A CNN processes data through convolution and pooling layers, where convolution layers extract features by applying filters and pooling layers reduce data dimensionality. Refs. [6,7] evaluate CNN with multichannel time-series data from body-worn sensors to achieve higher accuracy. The network has an input layer, convolutional layers, pooling layers, and fully connected layers, with the convolutional layer automatically extracting features and reducing input dimensions; pooling aims to preserve information while reducing complexity [8], and a fully connected layer combines features to make predictions.

### 2.1. Accelerometer and Inertial Data

Accelerometers are key sensors for HAR, because they provide measurements on acceleration over three axes. They can be placed in various body locations; however, the wrist generally offers the best recognition performance [9]. Achieving effective HAR involves signal preprocessing, feature extraction, and classification. In skateboarding, using Inertial-Magnetic Measurement Unit (IMU) data [10] achieved 97.8% accuracy in trick classification. Ref. [11] developed a mobile system with an IMU, detecting golf putts with high accuracy, while [12] explored jump frequency estimation in volleyball, but found the methodology ineffective. Still in volleyball, Ref. [13] used CNN to classify exercises, achieving over 90% accuracy. In skiing, Ref. [14] accurately classified exercise techniques using IMUs and ML. Ref. [15] predicted jumping errors with an IMU-CNN approach. Ref. [16] showed that IMUs readings could detect changes in a runner’s state with over 85% accuracy. Outlined standard procedures for HAR systems, complemented by a public dataset and MATLAB framework, were the focus of [17]. Achieving 96.7% accuracy [18] introduced a lightweight on-device learning algorithm.

### 2.2. Convolutional Neural Networks for Human Activity Recognition Using Mobile and Wearable Sensors

A challenging and still open aspect when dealing with HAR is the identification of the correct set of features for the classifier. Many studies focus on the feature extraction for HAR. In [19], the approach was based on online activity recognition on movement sensor data obtained from real-world smart homes, with four methods used to extract features from the sequence of sensor events and exploring both fixed static window size and dynamic varying window size. The results demonstrated that dynamic window sizes had the best performance.

Ref. [20] evaluated two different feature sets (time frequency and time domain) for HAR using data from 61 healthy subjects who performed seven daily activities—resting, upright standing, level walking, ascending and descending stairs, walking, uphill, and downhill—while wearing an IMU-based device. The device captured nine signals, including acceleration, angular rate, and Earth-magnetic field data, with a sampling frequency of 80 Hz. Each signal was segmented using a 5 s sliding window with a 3 s overlap. The study demonstrated that the time-frequency feature set consistently delivered higher accuracy.

CNNs combine feature extraction and classification, improving HAR performance, as shown in [6]. Health applications also explore HAR with wearable sensors; ref. [5] examined MCUs for health applications, with power consumption being a key challenge. Ref. [21] focused on pedestrian safety using wearable systems for accurate movement prediction. Ref. [22] proposed a memory compression technique for deploying Deep Neural Networks (DNNs) on resource-constrained MCUs, maintaining accuracy. The effectiveness of CNNs in HAR is highlighted in studies [7,23,24]. In [25] was demonstrated a 1D CNN model’s superiority in feature extraction, and [26] applied CNNs for sequence classification. In [27], Iss2Image was proposed as a method, converting sensor signals into images for CNN-based activity classification and outperforming existing methods. Neural Networks (NNs) and CNN have also shown success in sports classifications; ref. [28] compared MLs classifiers with NN for beach volleyball, while [29] implemented an NN for gesture recognition. A review [30] systematically evaluated NN for sport-specific movement recognition, with CNNs achieving high accuracy over other methods.

### 2.3. Human Activity Recognition Using Tiny Machine Learning

TinyML offers solutions for HAR on resource-limited devices; ref. [31] used transfer learning on MCUs, achieving high accuracy and low memory usage. Battery life and size constraints are major challenges for HAR systems on small devices, requiring low-power, lightweight ML models; ref. [32] optimized models to improve inference performance on MCUs, and ref. [33] found that model compression could maintain accuracy while reducing size by up to 10 times.

All in all, there are various methods to detect human activity recognition, but none have considered capturing movements off-the-person with great precision. Optical, or video, methods do not capture the precision an IMU achieves. Moreover, traditional wearable systems are on the person, being another item for the user to carry and very often colliding with the exercise execution.

## 3. Target Platform

**Figure 1:**
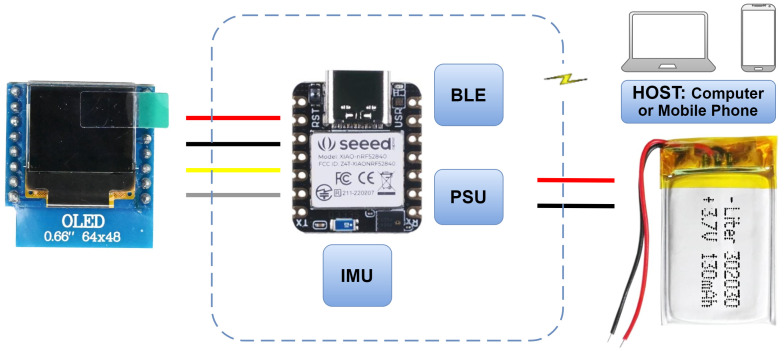
System architecture overview: Seeed Studio XIAO nRF52840 Sense MCU with IMU (6-axis), Power Supply Unit, and BLE.

The architecture of the system was defined to ensure low-power consumption, autonomy, and compact design. Each component was selected to maintain a balance between performance, size, and energy efficiency, given the limitations of running on a small LiPo battery and a small MCU. The Bluetooth Low-Energy (BLE) connectivity allows real-time data acquisition and sending the results to an external system, providing real-time feedback to the user. Moreover, the system architecture is designed with modularity in mind; see Figure 1. As new features or sensors are added, the MCU can be reprogrammed to accommodate these changes, making the platform versatile for future upgrades.

The MCU chosen is the Seeed Studio XIAO nRF52840 Sense, which features a Nordic nRF52840 CPU, 1 MB flash, and 256 KB RAM on chip memory. This compact MCU measures only 21 × 17.5 mm and weighs just 4 g. It also supports Bluetooth 5.0 to communicate with external devices. The embedded sensor LSM6DS3 IMU includes an accelerometer and a gyroscope.

For autonomous operation, a battery was incorporated into the system. A 302030 lithiumpolymer (LiPo) battery with a capacity of 120 mAh and 3.7 V voltage was chosen due to its small size, similar to the MCU. This battery connects directly to the power pins of the XIAO MCU.

Displaying the results of the real-time inference to the user is an important part of the system. A 0.66′′ OLED display with a resolution of 64 × 48 was selected. It is small, but fits the MCU size. The OLED display, like the battery, is powered directly by the Seeed Studio XIAO nRF52840 Sense. Additionally, a dedicated mobile application was developed to show the user the number of exercises performed and if they are correctly or incorrectly executed.

## 4. Methodology

This work was organized in two parts. The first one concerns the collection of raw sensor data from supervised execution of the exercises to create the dataset to train and test the neural network. The second part details the mapping of the neural network into the MCU software to classify the execution of the exercises in real time.

**Figure 2:**
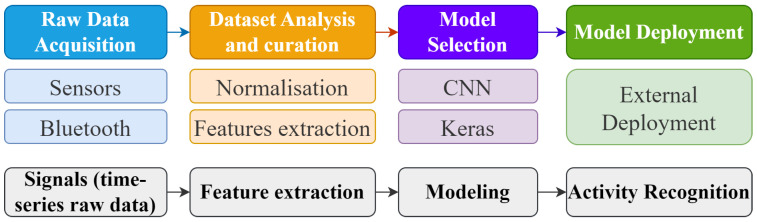
Tasks performed by the proposed human activity recognition system.

Both parts are performed with the same proposed HAR system, which is illustrated in Figure 2. There are 4 activities for training—raw data acquisition, data analysis and curation, model selection, and model deployment—and another 4 activities for the inference—signal acquisition, feature extraction, modeling, and activity recognition.

**Figure 3:**
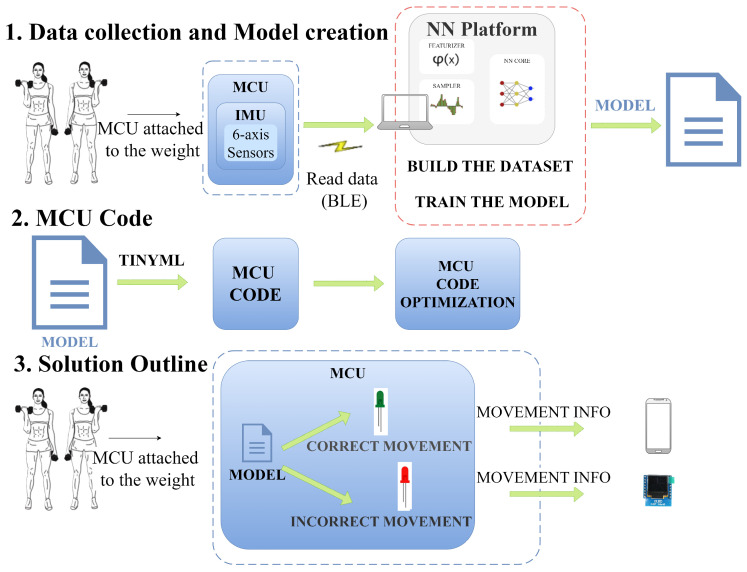
Solution outline.

The system collects movement data through the IMUs accelerometer and gyroscope. The MCU, equipped with a NN implementation, analyzes the movement data on the fly, categorizing movements as either well or poorly executed. This information is displayed on an OLED display and a mobile app. The steps are illustrated in Figure 3.

### 4.1. Raw Data Collection

Research on the topic revealed that there was no dataset available that could encompass the targeted exercises. Thus, it was mandatory to create a specific dataset. The input dataset to train the neural network was acquired using 6-axis sensors: accelerometer (X,Y,Z) and gyroscope (X,Y,Z). The then transmitted data wirelessly to the host computer via BLE. The accelerometer recorded in g’s, where 1 g is equivalent to the acceleration due to Earth’s gravity (9.81 m/s²) and gyroscope data recorded the angular speed in degrees per second, capturing the user’s movements. The X-axis indicates sideways or horizontal movement of the user, the Y-axis indicates upward or downward movement, and the Z-axis indicates forward or backward movement of the user.

### 4.2. Dataset Construction, Preparation, and Curation

The dataset was collected in a controlled environment at ISEL (Polytechnic of Lisbon) during a 4 h session with a total of 8 below-average athletes (age range: 19–57 years), with the expert supervision of a certified personal trainer. The dataset was limited to 8 subjects because it was being observed that the last ones did not introduce more variation into the dataset, and samples were very similar to the previous ones. The dataset includes data from various gym exercises; in total, 3 different exercises were performed correctly and incorrectly 10 times each by all the subjects: Shoulder Press, Bicep Curl, and Tricep Extension. The incorrect execution included different execution pace, exaggerated, and distorted movements, but still resembling the physical exercise.

**Figure 4:**
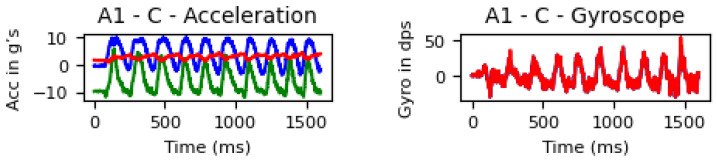
Signal acquired on the Bicep Curl exercise by the accelerometer (title Acceleration) and the gyroscope (title Gyroscope) from the 1 subject (X values in blue, Y in green, and Z in red).

**Figure 5:**
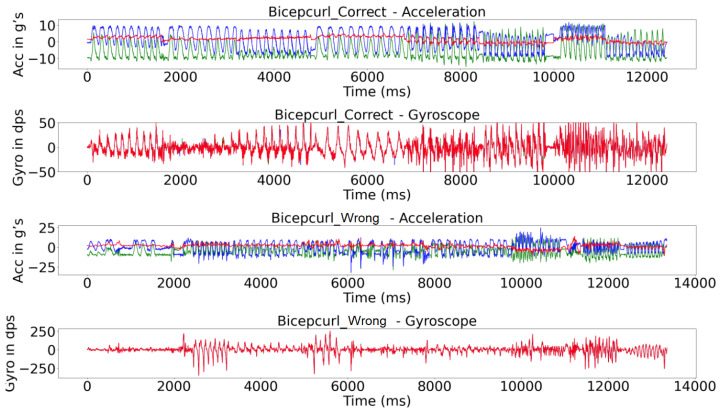
Signal acquired for the six exercises by all 8 subjects using the accelerometer and the gyroscope during 10 repetitions (X values in blue, Y in green, and Z in red. The bottom axis is represented by time (ms)).

The system was attached to the weight using a magnet to capture the activities at a sampling frequency of 50 Hz using the built-in tri-axial accelerometers and tri-axial gyroscopes; see Table 1. Figure 4 and Figure 5 show details of the acquired raw signal for the bicep exercise and all signals for all exercises by the 8 subjects, respectively.

Most activity classification methods use windowing techniques to divide the sensor signal into smaller time segments (windows). With the sliding window method, the signal is divided into windows of fixed length [34]. According to [35], mid-sized time windows (5 to 7 s long) perform best from a range of windows from 1 to 15 s for wrist-placed accelerometers. The results are slightly different for other accelerometer placements, but the trend of mid-size windows performing best remains a truth according to this study. The features extraction step involves calculating attributes that are able to capture the patterns over a sliding window; the features were extracted directly from the accelerometer raw data. According to [36], after testing multiple window sizes, they reach a conclusion that, in order to maintain a good trade-off between classification performance and resource utilization, a window of intermediate size (i.e., 3 s) has been proven to be the best; most of the times, 3 s was used. The window size was made in an adaptive and empirical manner to produce good segmentation for all the activities under consideration. After this investigation, a 3 s sliding window was used, with short or no overlap.

#### 4.2.1. Dataset Analysis and Curation

The first step in data preprocessing is to understand the data that were collected. Just looking at the dataset can give an intuition of what things were needed to be focused on. Applying NN training on noisy data would not give quality results as they would fail to identify patterns effectively. Data Processing is, therefore, to improve the overall data quality. Some of the examples are duplicates or missing values that may give an incorrect view of the overall statistics of data, or inconsistent data points that often tend to disturb the model’s overall learning, leading to false predictions. With this in mind, the next step was to clean the data.

Data Cleaning was performed as part of data preprocessing [37] to clean the data by filling missing values, smoothing the noisy data, resolving the inconsistency, and removing outliers. Removing noisy data involves removing a random error or variance in a measured variable, also removing the beginning and end of the collected samples, because those were times that the participant was stopped. All this information was removed manually. For normalization, the numerical attributes were scaled up or down to fit within a specified range. In this approach, the data were constrained to a particular set of values. The acceleration values were normalized to −4 to +4 and the gyroscope values to −2048 to +2047. Table 2 summarizes the number of samples and the duration for each exercise.

#### 4.2.2. Feature Extraction

Features are the values that result from processing raw-data that are fed into the neural networks. Meaningful features are extracted from the raw sensor data, such as statistical measures, frequency domain analysis, and others that may improve the model’s ability to distinguish between different activities.

### 4.3. Model Training

**Figure 6:**
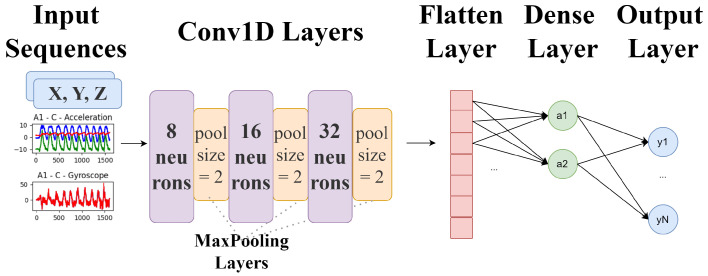
CNN model definition showing the included layers.

In this work, a three-layer stacked convolution and pooling was used for extracting features from the raw wearable sensor data (6 input signals), shown in Figure 6. Keras NN [38] is a library that simplifies the creation and training of NNs. Keras is the high-level API of the TensorFlow platform. Edge Impulse is an online platform based on Keras NN and uses TensorFlow Lite [39] for the training implemented with TFLite used the same approach.

The performance was measured using the following metrics: precision, ratio of correctly predicted positive observations to the total predicted positives for a class; recall, ratio of correctly predicted positive observations to all observations in the actual class; F1 Score, harmonic mean of precision and accuracy, providing a single metric that balances both; and accuracy, the count of predictions where the predicted value is equal to the true value. All these are illustrated in the following Equations (1)–(4) (from [31]) (TP = true positive rate; TN = true negative rate; FP = false positive rate; FN = false negative rate):

(1) $$\begin{array}{cc} precision & =\frac{TP}{TP\;+\;FP} \end{array}$$

(2) $$\begin{array}{cc} recall & =\frac{TP}{TP\;+\;FN} \end{array}$$

(3) $$\begin{array}{cc} F1 & =\frac{2\times precision\times recall}{precision\;+\;recall} \end{array}$$

(4) $$\begin{array}{cc} accuracy & =\frac{TP\;+\;TN}{TP\;+\;FP\;+\;FN\;+\;TN} \end{array}$$

#### 4.3.1. Edge Impulse

Edge Impulse is an online development platform for ML on edge devices [40]. It provides automation and low-code capabilities to make it easier to build datasets for edge devices and integrates with small portable MCUs. The platform builds datasets, train models, and optimize libraries to run on any edge device. Additionally, it provides a range of NNs architectures, including CNNs; it makes use of TensorFlow Lite for training, optimizing, and deploying deep learning models to embedded devices.

**Figure 7:**
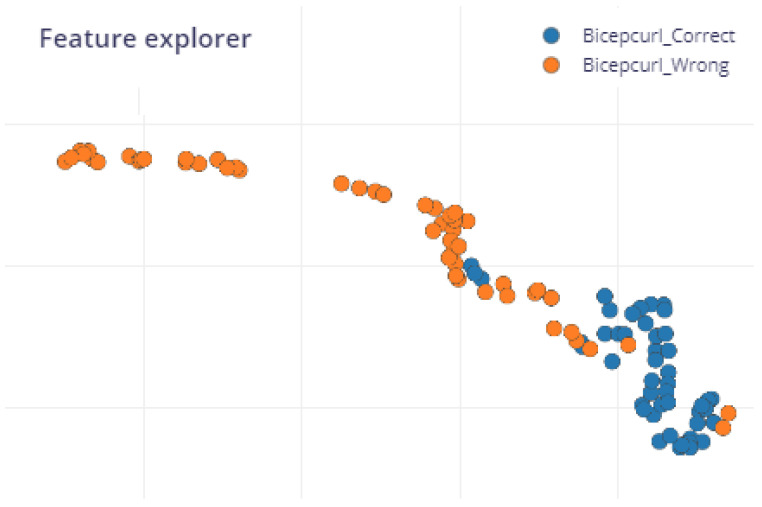
Edge Impulse: feature explorer using processing block spectral analysis.

In Figure 7, the data points for different classes (“Bicep_Correct” and “Bicep_Incorrect”) are in different colors, but there is overlap between the wrong and the correct class, probably because the movement wrongly performed was still very similar to the correct one. The built-in feature explorer was used to help analyze the dataset, the feature extraction (processing) method, and how the Machine Learning model will classify new samples.

A Convolutional 1D Network was used with the following CNN layers: three 1D Conv and three Max-Pooling, one Flatten, and two Dense Layers. The exercise “Bicep Curl” achieved an accuracy of 95%, the “Shoulder Press” achieved an accuracy of 94.1%, and “Tricep Extension” obtained an accuracy of 72.2%.

Edge Impulse also provides information about the implementation on the MCU. The inference time is 261 ms, the peak RAM usage is 62.1 kB, and the flash Memory usage is 348 kB.

#### 4.3.2. TensorFlow

The software platform used to build the system is based on Python 3.12.8 programming language combined with *Jupyter Notebooks* and an open-source library for artificial NNs called Keras running on top of TensorFlow. The sensor data were segmented using a 3 s sliding window with no overlap. The dataset consisted of six exercise classes with a total of 502 3 s windows, each containing 930 samples of both correct and incorrect variations.

Randomly split input and output pairs into sets of data: 60% for training, 20% for validation, and 20% for testing. To evaluate the accuracy of the model that was trained, some data are split to compare their predictions with real data and check how well they match.

- The training set is used to train the model.
- The validation set is used to measure how well the model is performing during training.
- The testing set is used to test the model after training.

The architecture of the CNN implemented using TensorFlow consists of an input layer, three convolutional layers with ReLUs and max-pooling, two fully connected layers, and a soft-max output layer for classification. The kernels applied are a small matrix used to filter input data in convolutional layers, while ReLU is an activation function that introduces non-linearity by outputting the input directly if it is positive; otherwise, it outputs zero.

The first hidden layer was a convolutional layer (Conv1D). In this layer, 8 filters were applied, with a kernel size of 3, for filtering each signal. Subsequently, the filtered output of the first convolutional layer was transformed non-linearly using ReLUs. The output of the ReLUs was then compressed using temporal max-pooling. After the temporal max-pooling, a second convolutional layer was applied. This layer took the transformed and pooled output of the first convolutional layer as input.

In the second convolutional layer, 16 filters were applied again to a 1D convolutional layer, kernels again with a length of 3 samples. The output of this layer was transformed and compressed by applying the ReLU non-linearity and overlapping temporal maxpooling into a third Conv1D layer. Following the two convolutional layers, fully connected ones were added, which took the non-linearly transformed and pooled output of the third convolutional layer as input. The fully connected layers (Dense) consisted of 16 and then 4 units, the units being the number of neurons or nodes, with the activation function set to ReLU.

The final output layer was designed to have as many neurons as gestures. For the output layer, the soft-max activation function was employed. Listing 1 shows the result of the training and its metrics.

**Listing 1.** Neural network training evolution.Epoch 1/600 − 2s 7ms/step − loss: 0.2635 − mae: 0.5057 − val_loss: 0.2401 − val_mae: 0.4868 … Epoch 600/600 − 1 s 5ms/s tep − loss: 2.6305 e−05 − mae: 0.0013 − val_loss: 0.3042 − val_mae: 0.357

Training Mean Absolute Error (MAE) represents the average absolute difference between the predicted values and the actual values on the training data. The validation loss is the error measure on the validation data, while validation MAE is the average absolute error on the validation data. Epochs are how many times our entire training set will be run thought the network during training.

The decrease in training loss from 0.2635 to 2.6305 × 10^−5^, and MAE from 0.5057 to 0.0013, across 600 epochs, indicates that the model has minimized the prediction errors. Meanwhile, the final validation loss and validation MAE values (0.3042 and 0.357, respectively) suggest that the model generalizes reasonably well. However, there may be slight over-fitting as the validation metrics are higher than the training metrics. The training and validation metrics should show similar results, indicating that the model performs consistently on both seen (training) and unseen (validation) data. When the training metrics are better than the validation metrics, it suggests that the model has learned the specifics of the training data too well, including noise or details that do not generalize to new data; this is characteristic of over-fitting.

Table 3 illustrates the confusion matrix for Bicep Curl correct and incorrect gesture. It should be noticed that the execution of the Bicep Curl movement is not very well discriminated—false positive (FP) is greater than true negative (TN). Thus, the output does not have a strong indication on the correctness of the movement. Such degraded levels of classification indicate that the dataset needs to be improved. Nevertheless, the correct execution of the exercise is predicted correctly with 92.31% for true positive (TP).

In the conversion of a TensorFlow model to a TFLite format (TensorFlow Lite format) using Keras API, it is saved as FlatBuffers, space efficient format, which was very helpful when the model needed to be deployed into the MCU. Then, the trained model has been optimized and converted into a C language representation by TensorFlow Lite framework [39]. To try to reduce the model size, it was applied after training quantization, which is a conversion technique that can reduce model size while also improving CPU and hardware accelerator latency, with some degradation in model accuracy. The full integer quantization [41] has benefits of 4× smaller and over 3× speedup. This technique of quantization reduces the precision of the numbers to fit in 8-bit integers. Afterwards, the generated model has been optimized and converted into TFLite format in order to be installed into the MCU.

## 5. Proposed Embedded System for Real-Time Exercise HAR

The implementation of a real-time embedded human activity recognition system used TensorFlow Lite in an MCU platform to run inference on the generated model. The system uses TensorFlow Lite for MCUs (TFLM) to run a pre-trained NN model that identifies if an exercise is well performed based on data from IMU sensors.

TFLite models are represented in a FlatBuffers format, which provides reduced size and faster inference compared with the TensorFlow’s Buffer format, due to its smaller code footprint and directly accessible data. The generation of the TFLite model can be performed through the following three different methods:

1 Using existing TFLite models from available examples;
2 Designing our own model through TFLite Maker; or
3 Converting TensorFlow models to TFLite using the TFLite converter and applying optimization method through the process.

In this work, option (3) was used, converting a TensorFlow model to TFLite using TFLiteConverter from the Keras [38] and Arduino, an open-source IDE to write a code and upload it to the board.

The proposed system is built around a MCU integrated with an LSM6DS3 IMU (LSM6DS3) IMU sensor. The LSM6DS3 sensor captures movement data, including acceleration and gyroscope measurements across the X-, Y-, and Z-axes, which are essential for recognizing different exercises. The work also uses TFLite, a lightweight version of TensorFlow designed specifically for running ML models on devices with limited computational resources.

An Organic Light-Emitting Diode (OLED) display shows feedback about the exercise and if it is correctly performed. To improve the user experience, the number of exercises correctly or incorrectly performed can be seen in the implemented mobile app in real time. The integration of the OLED display and mobile app improves the system interactivity and usability, making it easy for users to understand the system feedback immediately.

Once the data are collected, they are preprocessing to provide the features for the TensorFlow Lite model. This involves normalizing the raw acceleration and gyroscope data, ensuring that the data are suitable for the ML model to process. The preprocessed data are then input into the TensorFlow Lite model, which has been previously trained to recognize specific exercises. The model performs inference on the input data, using tensors, generating a set of probabilities that correspond to different predefined exercises. The exercise with the highest probability is identified as the recognized exercise. Finally, the system displays the exercise correctness on the OLED screen and mobile app, providing immediate feedback to the user.

The main application runs continuously, reading data from the IMU sensor, and preprocesses the required number of data samples, which are then input into the TensorFlow Lite model for inference. After the model processes the data, the recognized exercise is identified based on the highest output probability. The exercise is then displayed on the OLED screen, and in the Arduino Serial if connected to the PC. The application is designed to operate in real time, ensuring that the system remains responsive to new exercises and can provide immediate feedback to the user. This continuous monitoring and processing cycle makes the system effective for real-time movement recognition in multiple applications.

## 6. Android Mobile Application

A mobile application was developed to interface with the HAR system. The app provides users with a user-friendly way to monitor and track their exercises, displaying real-time feedback based on the data processed by the embedded system. For the app development, the OutSystems Low-Code Platform [42] was used. Low-Code is a visual software development approach that simplifies the creation of applications. The integration with the embedded system was made using BLE. The embedded system advertises that it is ready for connections. The BLE specification includes a mechanism known as notify that lets you know when data have changed. On the mobile app side, the connection is made to the embedded system, and afterwards, a listener is defined that is waiting for notification from the system, indicating that a movement was detected. A protocol of only 1 byte was defined to reduce the payload of the communication: 0 × 00—connected; 0 × 01—correct movement detected; 0 × 02—indicating an incorrect movement.

**Figure 8:**
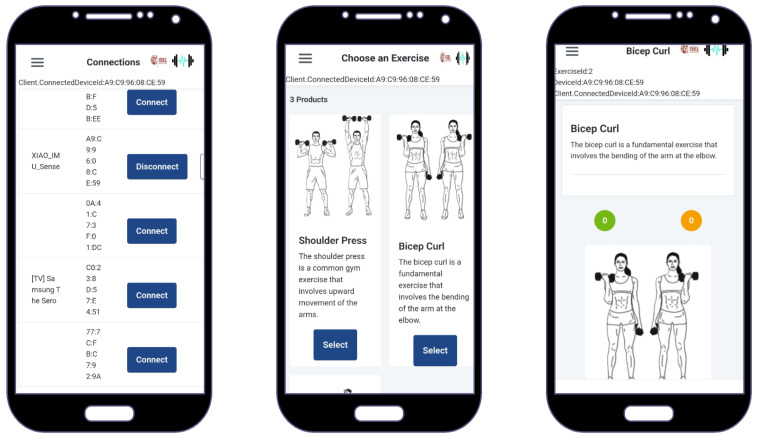
Mobile app: screens flow.

For the the proof of concept, 3 screens were designed and implemented, as in the following Figure 8:

- Connection screen—to connect/disconnect from the embedded system or do a test read;
- Exercises screen—to choose the exercise that will be performed;
- Exercise detail screen—to show the details of the chosen exercise and the counters: in green, the number of correctly made exercises, and in orange, the wrongly performed ones.

## 7. Experimental Results

The model trained on Edge Impulse achieved a good overall performance, with accuracy ranging from 72% to 95%, depending on the exercise performed. In Edge Impulse, the accuracy was between 70% up to 95%, while in TFLite, the accuracy was around 60%.

**Figure 9:**
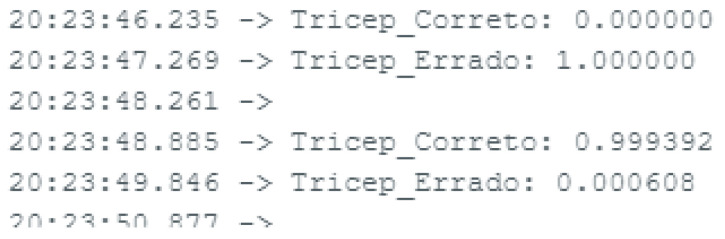
Snapshot of the console output showing the classified movement name and its probability.

The OLED display provides a user interface, which shows the recognized exercise immediately after its classification. The system’s performance can be further optimized by tuning the model, adjusting the sampling rate, or optimizing the TensorFlow Lite interpreter for specific hardware. Potential improvements include integrating more complex models, more exercises, or additional sensors for richer data input. The console output is shown in Figure 9. Table 4 and Table 5 show the results for the Edge Impulse and Python, respectively. Table 6 presents the comparison for the model size. The main conclusions that can be drawn from these results is that some movements have more variability in their execution; thus, they will lead to better discrimination between correct and incorrect execution. Moreover, results for the Edge Impulse platform are better than the CNN implementation in Python for raw data classification.

## 8. 3D-Printable Case

**Figure 10:**
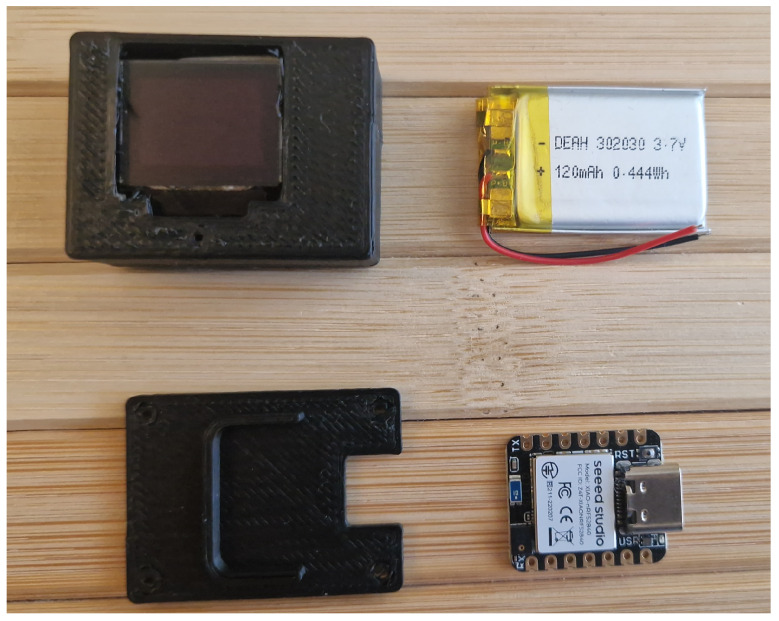
Prototype parts of the proposed system.

To assemble a prototype to be added to gym weights and indicating the user if the exercise was correctly performed, a box was built to make it a compact fully integrated systems, as illustrated in Figure 10. It is light (5 g), and all the components, including MCU, OLED, and the battery, are inside the box, making it completely portable.

The implemented embedded system for real-time movement recognition involves multiple components such as: MCU, an OLED display, a battery, illustrated in Figure 10.

## 9. Conclusions and Future Work

The goal of this work was to develop an autonomous embedded system to give the gym user feedback about the correctness of the exercise in real time. It also features a mobile application to register the classification outputs.

The final design of the proposed system is comprised of a low-power, low-cost, small-volume, and lightweight embedded system (less than 25 g) based on the MCU Seeed Studio XIAO nRF52840 Sense, a rechargeable battery, and an OLED display. These components were selected to obtain the best trade-off between performance, energy efficiency, and volume.

Since no existing dataset was found that could be re-used to fulfill the objectives of this work, there was a need to create a dataset tailored to the exercises in this study, under the supervision of a certified personal trainer.

Sample datasets were necessary for testing specific components of the system and the MCU, but collecting live data enabled the training of the NN to specific exercises chosen to this work, including both correctly and incorrectly performed. Regarding the size of the dataset, it was kept to a minimum to demonstrate the effectiveness of the proposed methodology without using very large datasets.

The classification algorithm implemented on the MCU uses NN techniques to train the model was created to be capable of categorizing movements based on their correctness and give that feedback in real time. It was trained using different platforms and techniques, including Edge Impulse and a reduced TensorFlow, TFLite model, optimized specifically for the hardware’s limited computational power and memory.

In comparison, Edge Impulse provides better accuracy, but it is limited to the features supported on the platform. TensorFlow can implement any NN model, but it takes longer to achieve good classification results.

The results indicated an accuracy of up to 94.1% in identifying exercises correctly or incorrectly performed. This validates the idea that real-time feedback can help on physical exercise safety.

All in all, in this paper, the applicability of the proposed system was demonstrated to prevent injuries from physical exercises. This work was limited to a small dataset of six exercises, so future research should explore a broader range of movements and incorporate larger datasets. Future work will focus on the classification method to improve the system’s accuracy and support for more and different exercises. Moreover, classification improvement can be achieved by investigating subject-dependent and subject-independent validations.

## Figures and Tables

**Table 1:** Summary of session used to acquire the raw data.

**Total duration**	4 h
**Subjects (female/male)**	8 subjects: 6 male, 2 female
**Range of ages**	19 to 57 years
**Skill level**	From beginner to professional athlete
**Sample rate**	50 Hz
**Sensor Placement**	On the Dumbbell
**Reference**	Manually labeled raw data
**Place**	ISEL Building F (DEETC)
**Accelerometer Resolution**	The accelerometer in the LSM6DS3 outputs data with a 16-bit resolution for each of the X, Y, and Z axes.
**Acceleration (m/s^2^)**	The constant 9.80665f (standard acceleration due to gravity on Earth) is used to convert the raw accelerometer data from units of g (where 1 g± = 9.81 m/s^2^) to m/s^2^.

**Table 2:** Correct and incorrect exercise execution dataset.

Bicep Curl Correct	Shoulder Press Correct	Tricep Extensions Correct
Data collected: 4 m 8 s 59 samples of 3 s each	Data collected: 4 m 8 s 59 samples of 3 s each	Data collected: 4 m 38 s 59 samples of 3 s each
**Bicep Curl Wrong**	**Shoulder Press Wrong**	**Tricep Extensions Wrong**
Data collected: 5 m 5 s 64 samples of 3 s each	Data collected: 4 m 2 s 36 samples of 3 s each	Data collected: 4 m 8 s 50 samples of 3 s each

**Table 3:** Confusion matrix for the classification of the Bicep Curl exercise.

Real∖Predicted	BicepCurl Correct	BicepCurl Wrong
**BicepCurl Correct**	92.31 (TP)	7.69 (FN)
**BicepCurl Wrong**	52.38 (FP)	47.62 (TN)

**Table 4:** Results obtained in the Edge Impulse platforms with the described CNN using the raw data.

Exercise	Precision	Recall	F1 Score	Accuracy (%)	Training Time (s)
**Bicep Curl**	0.80	0.80	0.80	80.0%	181
**Shoulder Press**	0.95	0.94	0.94	94.1%	181
**Tricep Extension**	0.73	0.72	0.72	72.2%	180

**Table 5:** Results obtained in the Python platform with the described CNN using the raw data.

Exercise	Precision	Recall	F1 Score	Accuracy (%)	Training Time (s)
**Bicep Curl**	0.76	0.65	0.64	65%	336
**Shoulder Press**	0.60	0.63	0.60	59%	350
**Tricep Extension**	0.60	0.60	0.60	60%	317

**Table 6:** Model size comparison.

Memory Size (KB)	Edge Impulse				TFLite			
	Bicep Curl	Shoulder Press	Tricep Extension	All	Bicep Curl	Shoulder Press	Tricep Extension	All
**Model Size**	N/A	N/A	N/A	N/A	256.8	256.7	256.4	256.8
**Quantized Model**	N/A	N/A	N/A	N/A	73.5	73.4	73.2	73.6
**TFLite Model**	348.0	348.0	348.0	348.0	453.7	453.0	451.8	453.9

## Data Availability

The original data presented in the study are openly available in GitHub at https://github.com/osduarte/TESE-MEIC-2324, see Appendix A.

## Abbreviations

AI	Artificial Intelligence
BLE	Bluetooth Low-Energy
CPU	Central Processing Unit
CNN	Convolutional Neural Network
DNN	Deep Neural Network
FBT	Full-Body Tracking
GPU	Graphics Processing Unit
HAR	Human Activity Recognition
IMU	Inertial-Magnetic Measurement Unit
IoT	Internet of Things
LiPo	lithium-polymer
LSM6DS3	LSM6DS3 IMU
MAE	Mean Absolute Error
MCU	Microcontroller Unit
MEM	Micro Electro-Mechanical System
ML	Machine Learning
NN	Neural Network
OLED	Organic Light-Emitting Diode
PRA	Peak Resultant Acceleration
PVA	Peak Vertical Acceleration
SRAM	Static Random-Access Memory
TinyML	Tiny Machine Learning
TFLite	TensorFlow Lite
TFLiteLibrary	TensorFlow Lite Library
TRL	Technology Readyness Level

## Appendix A. Source Files

The work developed is publicly available under MIT Open Source License on a repository, where all the implementation details and supplementary materials are stored. The repository can be found in https://github.com/osduarte/TESE-MEIC-2324 (accessed on 11 December 2024).
